# A Longitudinal Empirical Study on the Association Between Urban Green Space Ratio and Population Health Indicators

**DOI:** 10.3390/healthcare13101109

**Published:** 2025-05-10

**Authors:** Wen Zhou, Jie Xu, Yiqi Yu

**Affiliations:** College of Horticulture and Landscape Architecture, Yangzhou University, Yangzhou 225000, China; xj8650@126.com (J.X.); yuyiqi1717@163.com (Y.Y.)

**Keywords:** urban green space, comprehensive health level, local climate zone, quantile regression

## Abstract

**Background**: The positive effects of urban green space (UGS) on public health and well-being have been confirmed. However, most previous studies on the health benefits of UGS have focused on the influencing factors, mechanisms, and different groups of people, with little attention paid to regional heterogeneity. **Methods**: Using provincial-level panel data from China (2007–2020), this study measures residents’ comprehensive health levels (CHLs) through factor analysis encompassing physiological, mental, and social dimensions. Fixed-effects models and panel quantile regressions are employed to analyze UGS–health associations across climatic zones and health status quantiles. **Results**: The CHL of residents in China has improved as a whole, but with some provinces showing a declining or unpredictable trend. The results of the effects of UGS on the health status of urban residents were inconsistent. Overall, the amount of UGS is positively related to the CHL of the inhabitants (Coef. = 0.113; *p* < 0.01). In addition, the health-promoting effect of UGS is significantly stronger in provinces with a higher health level than in provinces with a lower health level, and no positive effect was observed in the provinces with the lowest health level. Increasing the amount of UGS can effectively improve the CHL of residents in the mid-temperate (Coef. = 0.189; *p* < 0.05) and warm temperate (Coef. = 0.135; *p* < 0.05) regions, but no health-promoting effect was found in the subtropical regions. **Conclusions**: This study expands our scientific understanding of the effects of UGS on the comprehensive health status of urban residents.

## 1. Introduction

More than half of the world’s population lived in urban areas in 2018, and that proportion is projected to rise to around two-thirds by 2050 [1]. In China, more than 65% of the population lived in urban areas in 2022 [2], and this is expected to reach 75% by 2050 [3]. Urbanization is accompanied by many environmental hazards such as increased traffic, air pollution and noise pollution, as well as an intensified urban heat island (UHI) effect [4,5]. The most obvious landscape change associated with rapid urbanization is the replacement of many natural surfaces (e.g., urban green spaces and water bodies) with impervious surfaces and built-up areas [6,7]. The decrease in the amount of urban green space and the increase in population density in cities have led urban dwellers to have less and less contact with the natural environment. These changes and processes pose a major challenge to the functioning of global ecosystems and to human health and well-being [8].

There is growing evidence that many chronic diseases such as diabetes, obesity, and depression are linked to a sedentary lifestyle and chronic stress [9]. In China, the proportion of deaths caused by chronic diseases has reached over 85%, and the medical treatment rate for chronic diseases was nearly 30% in 2018 [10]. In addition, 73.6% of urban residents were in a state of mental sub-health, with 16.1% of urban residents having mental health problems of varying degrees, while only 10.3% of urban residents were completely mentally healthy from 2012 to 2017 [11]. The population suffering from chronic diseases and mental health problems accounts for a large proportion in China. Many of these health problems can be prevented through social interventions, such as investing in a healthy environment for physical activity and recreation [12]. The increasing interest in healthy lifestyles and better health outcomes has recently led to numerous studies investigating possible links between the natural landscape and human health, particularly in urban environments [13,14,15].

Previous studies have demonstrated that urban green space is a nature-based solution to challenges of urbanization and health promotion [16,17,18]. Urban green spaces provide space for physical activity and social interaction for city dwellers [19,20] and enable psychological recovery (relieving the pressures of life and work for residents in a high-density urban environment) [21,22], which has a direct positive impact on physical, social, and mental health. In addition, urban green spaces provide environmental benefits by mitigating the UHI effect [23,24] and reducing air pollution [25,26], thereby improving the living environment and health level of urban residents. For example, unfavorable pregnancy outcomes have been shown to be related to environmental factors such as poor air quality [27] and high temperatures [28], which can be regulated by urban green spaces. The studies by Kim et al. [29] and Sbihi et al. [30] found that the density of street trees and other urban green spaces is associated with less obesity and asthma in children. In addition, the health of older people can particularly benefit from access to urban green spaces [31], as they are the largest user group [32]. Overall, the direct and indirect positive impacts of green spaces on human health have been continuously confirmed. Previous research has primarily focused on the impact of green spaces on specific populations or diseases, with relatively fewer studies examining their effects from a macro-scale perspective—particularly in comparing regions with varying health levels and climatic conditions.

To address these insufficiencies, we conducted an empirical research study using panel regression models and spatial autocorrelation analysis, based on China’s provincial panel data from 2007 to 2020, to (1) assess the spatio-temporal changes and clustering of the CHL and per-capita park green area (PGA per capita) of urban residents; (2) determine the impact of PGA per capita on CHL at different health levels; and (3) investigate the effect of the climatic background on the relationship between CHL and PGA per capita.

## 2. Data and Methods

### 2.1. Data Descriptions

**Figure 1:**
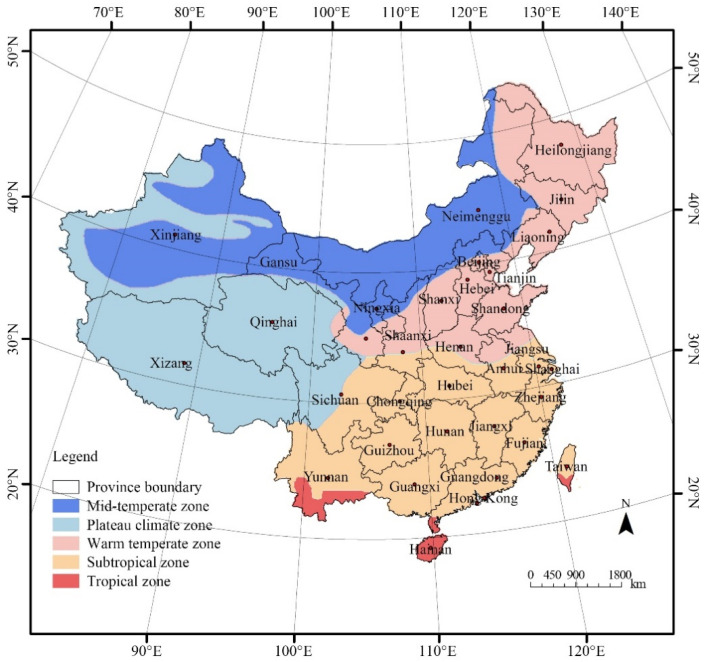
Geographical maps of China showing the locations of provinces and five major climatic zones.

This study includes panel data of urban residents in 31 provincial units in mainland China from 2007 to 2020, including 22 provinces, 5 autonomous regions, and 4 municipalities directly under the central government (Figure 1). For convenience, the term “province” is used uniformly in the following text. The data used in this study were extracted from the China Health Statistics Yearbook [10], China Statistics Yearbook [2], China Civil Affairs’ Statistical Yearbook [33], and Procuratorial Yearbook of China [34].

### 2.2. Methods

#### 2.2.1. Factor Analysis

Factor analysis was performed to calculate the Comprehensive Health Index (CHI) of residents using SPSS 23.0. The selection of indicators was mainly based on data availability, the current health status of Chinese residents, and prior research. As defined by the WHO, health is a state of complete physical, mental, and social well-being and not merely the absence of disease or infirmity [35]. Thus, indicators to measure the CHI were selected from three dimensions in this study: physiological, mental, and social, to measure the comprehensive health level of the residents. Specifically, life expectancy, perinatal mortality, maternal mortality, the incidence rate of Class A and B notifiable infectious diseases, the morbidity rate of Class A and B notifiable infectious diseases, and the number of medical outpatients were selected as physiological measurement indicators [36,37,38]. In addition, the emergency rate of psychiatric departments, the crude divorce rate, and the crime rate were selected as the evaluation indicators from mental and social perspectives. Previous studies have demonstrated that numerous social determinants of health are associated with the crude divorce rate and the crime rate [39,40,41,42]. The specific definitions can be found in Table 1.

Due to the different attributes and dimensions of the various indicators, it is necessary to process each indicator individually before performing a factor analysis for panel data. First, we normalized the reversed indicators (i.e., *X*_2_, *X*_3_, … *X*_9_) using the reciprocal method as they all make a negative contribution to the comprehensive health level. Subsequently, the Z-score standardization method was applied to eliminate dimensional problems between the data. Finally, the KMO (Kaiser–Meyer–Olkin) test and Bartlett’s test were used to determine the feasibility of the factor analysis. The results showed that the KMO value was 0.735 (greater than 0.6), and the approximate chi square value (χ^2^) of Bartlett’s test of sphericity was 2573.487 (*p* < 0.05) (Table 2), indicating significant correlation between the variables (*X*_1_, *X*_2_, … *X*_9_) and suitability for subsequent factor analysis.

Table 3 shows the estimated values derived from principal component analysis (PCA). A total of three components were generated, accounting for 72.6% of the total variance. This indicates that PCA can effectively preserve information and reduce redundancy in the original dataset. Component 1 explains 33.7% of the total variances. As shown in Table 4, the emergency rate of psychiatric departments and crude divorce rate exhibited high loading coefficients on Component 1, and were defined as mental health indicators in this study, while Component 2 accounted for 27.5% of the total variances. Variables with high loadings on this component included life expectancy, perinatal mortality, maternal mortality, the incidence rate of Class A and B notifiable infectious diseases, the morbidity rate of Class A and B notifiable infectious diseases, and the number of medical outpatients. These were classified as physiological health indicators. Component 3 contributed 11.4% of the total variance, with the crime rate showing a high loading coefficient. This variable was designated as a social health indicator.

The factor score coefficient matrix obtained by the regression method is shown in Supplementary Table S1, and the score functions of each factor can be obtained. Next, the ratio of the variance contribution rate of each rotated factor to the cumulative variance contribution rate was used as the weight for calculating the CHI of residents.

#### 2.2.2. Fixed-Effects Model

The analytical framework is based on Grossman’s theory of health production [43]. This theory states that the health of residents is a commodity produced by a series of input factors such as lifestyle, living environment, education, income level, and medical services. Therefore, the following production function was established:

Health = f(urban green space, economic level, medical services, social development, education level)

The empirical analysis of the panel data for this study was conducted using Stata 18.0. Based on the results of the F-test and Hausman test, a fixed-effects (FE) model was applied to quantify the impact of UGS on residents’ CHI instead of random effects and pooled OLS models. In the FE model, the CHI was used as the dependent variable. The amount of urban green space is the key explanatory variable, and park green area per capita (PGA per capita) was chosen in this study. In addition, relevant indicators of economic, medical, social, educational, and health factors that may affect the health of residents were selected as control variables. Specifically, GDP per capita (GDP per capita) was chosen as a measure of the economic level; the number of medical and health institutions was chosen as an indicator to measure regional medical resources, using the word “medical” for simplicity; the permanent population and urbanization rate were chosen as social indicators; and education expenditure per capita (education) was chosen to measure the local education level.

The panel data were strong balanced, and the Z-score standardization method was applied to eliminate dimensional problems between different variables. The regression model for the panel data was established as follows:

$$\begin{array}{c} CHI_{it}=\alpha_{i}+\beta_{1}PGApercapita_{it}+\beta_{2}GDPpercapita_{it}+\beta_{3}Medical_{it}+\beta_{4}Permanent\;population_{it}\\ +\beta_{5}Urbanization\;rate_{it}+\beta_{6}Education_{it}+\varepsilon_{it} \end{array}$$

where *i* and *t* represent various observed individuals and times, *α_i_* is the individual effect, representing the impact of unobserved variables in the model on individual differences in the cross-section, *β* is the coefficient, and *ε_it_* is a random perturbation term.

#### 2.2.3. Panel Quantile Regression Model

Panel quantile regression models (QRPD) refer to the analysis of the marginal influence of a variable on the CHI in each quantile, based on the average influence of the independent variable on the dependent variable and further assuming values between 0 and 1. In this study, QRPD were created for the 10%, 30%, 50%, 70%, and 90% quantiles. This allows a more comprehensive description of the conditional distribution of the explained variables and a combination of the above theory to investigate the degree of impact of urban green spaces on residents’ health at different health levels. This article chooses the panel quantile regression model to construct relevant empirical models and constructs the following model form:

$$\begin{array}{c} Q_{{CHI}_{it}}(\tau |\chi_{it},\alpha_{i})=\alpha_{i}+\beta_{1\tau }PGApercapita_{it}+\beta_{2\tau }GDPpercapita_{it}+\beta_{3\tau }Medical_{it}+\\ \beta_{4\tau }Permanent\;population_{it}+\beta_{5\tau }Urbanization\;rate_{it}+\beta_{6\tau }Education_{it}+\varepsilon_{it} \end{array}$$

where *i* and *t* stand for various observed individuals and times, *τ* is the value of the percentile, *α_i_* is the individual effect, representing the impact of unobserved variables in the model on individual differences in the cross-section, *β* is the coefficient, and *ε_it_* is a random perturbation term.

#### 2.2.4. Grouped Regression Model

To explore the regional heterogeneity of the health-promoting effect of UGS and clarify whether the impact of UGS on the CHI is consistent under different climatic conditions, panel grouped regression analysis was further performed. There are five main climate types in China: the mid-temperate climate, plateau climate, warm temperate climate, subtropical climate, and tropical climate. Specifically, there are 4 provinces in the mid-temperate zone, 2 in the plateau region, 9 provinces in the warm temperate zone, 11 provinces in the subtropical zone, and 1 in the tropical zone. As the number of provinces in the plateau region and in the tropical zone is less than 3, which does not fulfil the criteria for a panel regression, these two climate types were not included in the grouped regression category. Therefore, 24 provinces with 3 climate types were included in this grouped regression analysis.

#### 2.2.5. Spatial Autocorrelation Analysis

Global Moran’s I was used to test whether the CHI and PGA per capita of 31 provinces in China were spatially autocorrelated and to what extent they were correlated. The value of Global Moran’s I ranges from 1 to −1, indicating complete spatial clustering to spatial dispersion. A larger positive value indicates a stronger positive spatial correlation and a smaller negative value indicates a greater spatial dispersion. When Moran’s I value approaches 0, it is defined as a spatial random distribution. In addition, the local Moran’s I is applied to identify different types of spatial clustering or dispersion, such as High–High, High–Low, High–Low, and Low–High clusters for CHI and UGS.

## 3. Results

### 3.1. General Information of CHI and PGA per Capita

**Figure 2:**
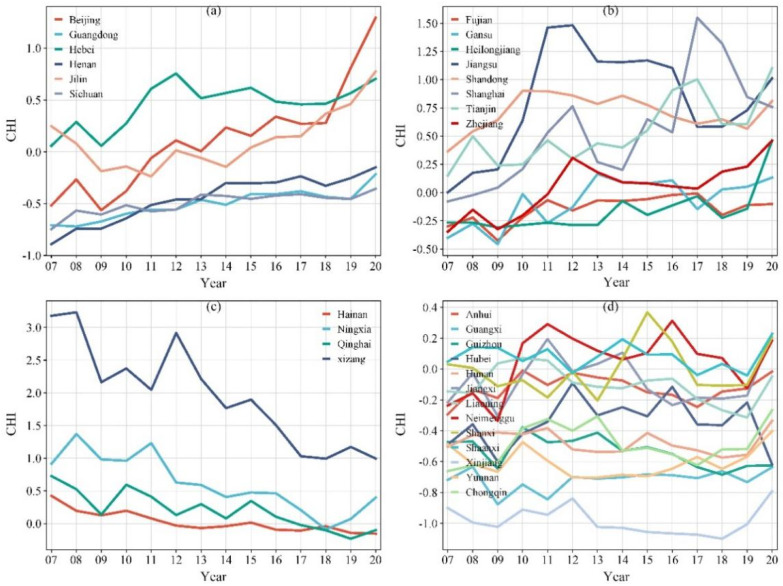
Four annual trends in the CHI of 31 provinces in China from 2007 to 2020: (**a**) Continuous increase; (**b**) Fluctuating upward trend; (**c**) Decrease; (**d**) Unpredictable pattern. Note: CHI stands for Comprehensive Health Index; 07 stands for 2007, 08 for 2008, and so on.

**Figure 3:**
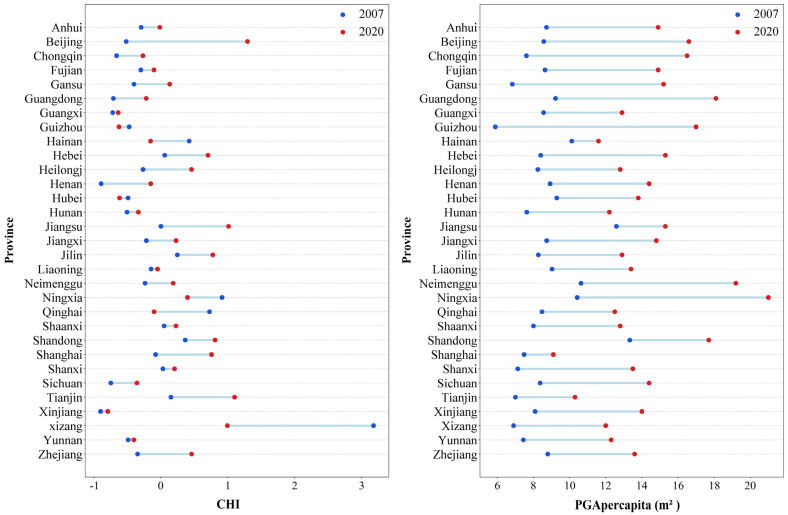
Variations in CHI and PGA per capita of 31 provinces from 2007 to 2020. Note: CHI stands for Comprehensive Health Index, PGA per capita for park green area per capita.

As shown in Figure 2, the results of this study suggested that the temporal fluctuations of the residents’ CHI differed considerably between the individual provinces. In some provinces (e.g., Beijing, Guangdong, Hebei, etc.), the CHI continuously increased, while in other provinces, such as Hainan, Ningxia, Qinghai, and Xizang, it decreased from 2007 to 2020. Moreover, a fluctuating upward trend and an unpredictable trend were also observed in some provinces. The change ranges of the CHI between 2007 and 2020 were also very different in the various provinces (Figure 3). More specifically, Beijing’s CHI rose from the 25th to the 1st place, Tianjin’s from the 7th to the 2nd, and Jiangsu’s from the 11th to the 3rd place. Meanwhile, Qinghai’s CHI fell from the 3rd to the 19th place, and Hainan’s from the 4th to the 22nd place. From 2007 to 2020, the PGA per capita increased in all provinces, but the growth rate was significantly different. For example, the PGA per capita in Hainan and Shanghai only increased by 1.48 m^2^ and 1.62 m^2^, respectively. However, it increased by 11.11 m^2^, 10.58 m^2^, and 8.88 m^2^ in Guizhou, Ningxia, and Guangdong, respectively.

**Figure 4:**
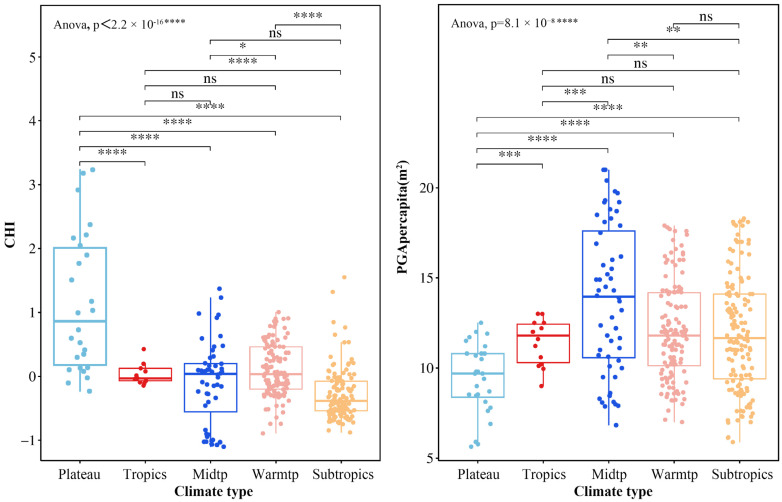
The distribution of CHI and PGA per capita. Note: *, **, ***, and **** indicate significantly different means based on an independent-sample *t*-test at 0.05, 0.01, 0.001, and 0.0001 levels, respectively. CHI stands for Comprehensive Health Index, PGA per capita for park green area per capita, Midtp for mid-temperate, Warmtp for warm temperate, and ns for no significant difference between groups.

There were significant differences in the CHI and PGA per capita between provinces with different climatic backgrounds (Figure 4). The values of the CHI of the provinces in the plateau climatic zone were significantly higher than in the other provinces, and they were lowest in the subtropics. In addition, the provincial heterogeneity of the CHI was highest in the plateau climatic zone, followed by the mid-temperate and warm temperate zones, and lowest in the subtropics and tropics. In addition, the PGA per capita was highest provinces in the mid-temperate zone, followed by the warm temperate, subtropical, and tropical zones, and lowest in the plateau climatic zone, and the provincial heterogeneity of PGA per capita followed a similar pattern.

### 3.2. Spatial Autocorrelations of CHI and PGA per Capita

**Figure 5:**
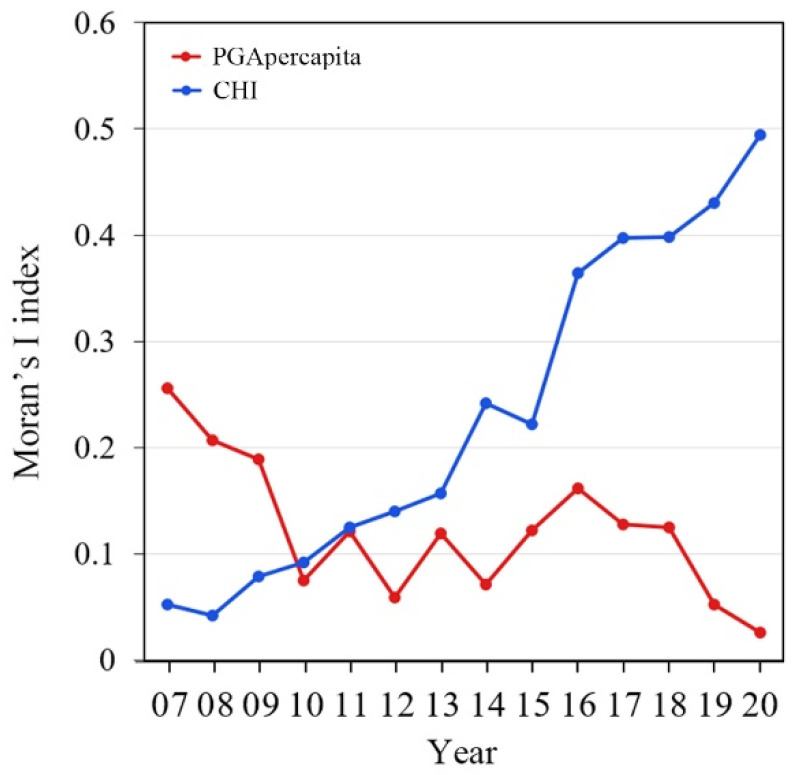
Annual variations in spatial correlation of CHI and PGA per capita from 2007 to 2020.

As shown in Table 5, the results of the spatial autocorrelation revealed statistically significant Global Moran’s I values for the years from 2011 to 2020, suggesting that the global spatial distributions of the CHI of China’s residents were positively autocorrelated in these years. The Global Moran’s I values were all greater than 0 (*p* < 0.05), indicating a clear spatial clustering pattern in the distribution of the CHI. After 2011, the spatial autocorrelation showed an upward trend over time, reaching its highest value in 2020 (Figure 5). In early years (e.g., 2007–2010 in this study), no significant Moran’s I values were observed. Similarly, there were positive autocorrelations of the spatial distribution of PGA per capita in China (Global Moran’s I > 0, *p* < 0.05) in most years. No significant Moral’s values of PGA per capita were observed in 2010, 2012, 2014, 2019, and 2020. In contrast to the CHI, the spatial autocorrelation of PGA per capita in China showed a downward trend over time, and the value was highest in 2007 (Figure 5).

**Figure 6:**
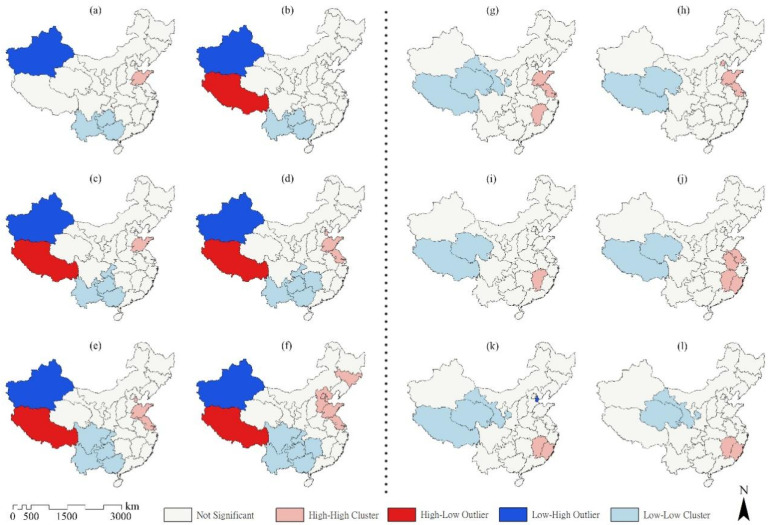
LISA cluster map of local autocorrelation analysis of CHI in (**a**) 2011, (**b**) 2012, (**c**) 2014, (**d**) 2016, (**e**) 2018, (**f**) 2020, and PGA per capita (**g**) 2007, (**h**) 2009, (**i**) 2011, (**j**) 2013, (**k**) 2016, and (**l**) 2018 among 31 provinces in China.

As the LISA (local indicators of spatial association) map shows (Figure 6), there are four spatial cluster types in China, High–High, High–Low, Low–High, and Low–Low, for CHI, and mainly two types, High–High and Low–Low, for the PGA per capita. Xinjiang was a Low–High clustering area of CHI from 2011 to 2020, and Xizang was a High–Low clustering area from 2012 to 2020. In addition, southern provinces such as Yunnan, Guangxi, and Guizhou were Low–Low clustering areas, and the Low–Low clustering areas expanded after 2012. By 2020, most southern provinces, including Yunnan, Guangxi, Guizhou, Hunan, Chongqing, and Sichuan, were Low–Low clustering areas. Jilin was a High–High clustering area in 2011, and many eastern provinces, including Shandong, Jiangsu, Tianjin, Beijing, and Hebei, turned into High–High clustering areas of the CHI in 2020. Western areas of China, including Xizang, Qinghai, and Gansu, were Low–Low clustering areas of PGA per capita. Meanwhile, the High–High clustering areas shifted from the east (e.g., Shandong and Jiangsu in this study) to the southeast of China (e.g., Jiangxi and Fujian in this study) from 2007 to 2018. Overall, the results indicated that the correlation between the CHI and spatial distribution is increasing, while the correlation between PGA per capita and spatial distribution is weakening.

### 3.3. The Influence of Green Spaces on the Comprehensive Health Level of Residents and Regional Heterogeneity

In this study, panel quantile regression models (QRPD) were created for the 10%, 30%, 50%, 70%, and 90% quantiles and are presented together with the regression results of the fixed-effect (FE) panel model in the following table (Table 6). The variance inflation factor (VIF) values ranged from 1.02 to 1.36, with all 1/VIF values exceeding 0.73, indicating a low degree of collinearity among the variables in this study (Supplementary Table S2). Overall, the estimated values and signs of the coefficients of the variables varied depending on the health level of the residents. The results indicated that there are differences in the effects of the variables on the CHI for different health levels of residents. According to the FE panel regression results, the GPA per capita (*p* < 0.001), GDP per capita (*p* < 0.001), medical (*p* < 0.001), population density (*p* < 0.001), and education (*p* < 0.005) all had significant positive effects on residents’ CHI, while the permanent population had a negative effect (*p* < 0.001). According to the results of QRPD analysis, the estimated coefficient of the GPA per capita was positive at all quantiles except the 10% percentile, and the estimated coefficient increased as the quantiles increased. With the exception of the estimated coefficients at the 30% and 50% percentiles, which were smaller than those in the fixed-effects model, the estimated coefficients at the other percentiles were larger than those in the fixed-effects model.

Based on the results of the FE panel regression, all variables together could explain 44.4% of the variation in the CHI. By taking into account the individual differences caused by the climatic background, the fitting degree was considerably improved. Specifically, the GPA per capita and permanent population together could explain 83.1% of the variation in the CHI in the mid-temperate provinces; the PGA per capita, GDP per capita, medical, population density, and permanent population could explain 56.4% in the warm temperate provinces; and the GDP per capita and permanent population could explain 59.2% in the subtropical provinces. The results of the heterogeneity analysis showed that the impact of the PGA per capita on provinces with different climatic backgrounds was inconsistent. In particular, the estimated value of the PGA per capita was higher in the provinces with a mid-temperate climate than in the provinces with a warm temperate climate, and both were higher than the results of the FE panel regression. The PGA per capita had no effect on the health level of residents in the subtropical provinces, or none that could be detected by the FE panel regression analysis alone.

By using the method of replacing the core explanatory variable, that is, replacing the PGA per capita with the green coverage rate of built-up areas, the above model was reconducted and similar results were still obtained (Supplementary Table S3), confirming the robustness of the regression model results.

## 4. Discussion

From 2007 to 2020, the health status of residents in most provinces improved, with the overall trend being high in East China and low in Southwest China. The trend of comprehensive health level changes over time could be divided into four categories: (1) gradual increase (e.g., Beijing, Guangdong, Hebei, etc.); (2) fluctuating increase (e.g., Shanghai, Jiangsu, Zhejiang, etc.); (3) decline (e.g., Hainan, Ningxia, Qinghai, and Xizang); and (4) unpredictable patterns (e.g., Anhui, Guangxi, Guizhou, etc.). Unlike the CHI, the per-capita park green area in all provinces showed a consistent upward trend in the past 14 years, and the per-capita park green area in China increased by 5.73 m^2^. Residents in provinces with a relatively large per-capita park green area (Beijing and Shandong) also had a higher overall health level. However, some provinces with a high comprehensive health level of residents, such as Tianjin, Xizang, and Jilin, ranked at the bottom in terms of PGA per capita. The results indicate that the per-capita park green area affects the health level of residents, but it is not the only influencing factor. This study has demonstrated that the health level of residents is highly correlated with factors such as the local population, income level, urbanization rate, medical resources, and education level, and the correlation shows regional heterogeneity.

Increasing urban green spaces contributes to improving the comprehensive health level of residents, which is consistent with the conclusions of previous research [25,44,45,46]. In addition, we found that the impact of the per-capita park green area on residents’ comprehensive health level varies by region. In provinces with better health conditions, the health-promoting effect of urban green space is stronger. This is probably because when the comprehensive health status reaches a certain level, people are more willing to go out and use urban green space, and they benefit from the positive effects of urban green space in physiological, psychological, and social aspects through its mediation and regulation mechanisms (reducing thermal environment, lowering urban noise, reducing air pollution, encouraging residents to engage in physical exercise, promoting social cohesion, etc.). Moreover, the economic level has no influence on the health level of these areas, while the improvement of medical and educational levels also contributes to the improvement of the comprehensive health level of the inhabitants in these areas. In provinces with a weaker health status, the effect of green areas on health is relatively weak. In the provinces with the weakest comprehensive health condition, no health-promoting effect of urban green spaces was found. Therefore, for different provinces, it is necessary to develop reasonable strategies for urban green space construction based on the actual health level of residents, especially in areas with better health conditions, and to further leverage the health promotion benefits of urban green spaces. For areas with poor health levels among residents, it is even more important to fully leverage the positive impact of factors such as the economy and healthcare on residents’ health in order to improve their overall health.

In this study, a grouped panel regression model was used to further investigate the effects of green space on residents’ health under different climatic conditions, and it was confirmed that the climate can influence the effects of urban green space on the comprehensive health level. The fitting coefficients of the three grouped panel regressions were all greater than the results of the fixed panel regression, which means that climate units can describe more individual differences when compared with provincial units. Compared with other climate zones, urban green space is the most important determinant of residents’ health in mid-temperate provinces. Therefore, increasing the quantity and quality of urban green spaces in mid-temperate provinces can effectively improve the health level in this region. In contrast, economic, medical, and educational levels have no influence on health. The effects of all variables on the provinces in the warm temperate zones are essentially the same as at the national level, and urban green space, the economy, healthcare, and education all have an impact on the health level of the regions’ inhabitants. In the subtropical regions, the increase in the amount of urban green spaces has no significant effect in improving the comprehensive health level of the region’s inhabitants, while the medical level has a significant effect. This is probably because the comprehensive health level of the provinces in this region is significantly lower than those in other climate zones, which is the reason for inhabitants’ low demand for a high-quality ecological environment.

There are regional differences in the impact of the urbanization level on the health of inhabitants, and the influence of regional differences should also be taken into account when introducing corresponding policy measures in China. Overall, the positive effects of urbanization on the health of residents far outweigh the negative consequences. A high degree of urbanization gives residents access to better medical care and education, increases their employment opportunities and income levels, and thus improves their state of comprehensive health [47]. On the other hand, urbanization can bring problems such as environmental pollution, more work pressure and fast food, and fewer opportunities for physical activity, thereby increasing the probability of residents suffering from obesity and hypertension and worsening their health [48]. In regions with different health statuses, the urbanization level has a health-promoting effect on residents. Furthermore, the effect of the urbanization level is significantly greater in regions with a better state of health of inhabitants than in regions with a poorer health state of inhabitants. The impact of the education level on health is the weakest among the selected indicators. In areas with poor health levels, the improvement of education has a negative impact on residents’ health. This may be because the improvement of the education level leads to higher social expectations and competitive pressure in the workplace, while long-term high pressure, prolonged sitting, and irregular sleep patterns may lead to chronic diseases (such as cardiovascular disease, cervical spondylosis) or psychological problems (anxiety, depression), worsening the health of residents in areas with poor health levels.

In provinces with high comprehensive health levels and in regions with mid-temperate and warm temperate climates, efforts should be made to increase green spaces near residential areas or to optimize public facilities in existing green spaces in order to increase the frequency of use of urban green spaces by residents and improve their social interaction function. In addition, the existing vegetation types and spatial functions can be more appropriately designed or optimized based on the characteristics of local residents, so that the green spaces can better deliver their ecological and health benefits. For example, if the residents in the area are mainly elderly people, some plants with curative effects should be planted or exercise and leisure facilities should be designed and added. In provinces with an overall poor health status of residents, the development and optimization of urban green spaces need not be considered when it comes to improving the comprehensive health status of the region. Instead, measures such as improving the economic level of the region to increase the income of residents, increasing the allocation of medical resources, and increasing education spending can be taken to improve the comprehensive health level. In the subtropical regions, decision-makers should pay more attention to improving the level of urbanization and the income level of residents.

Although this study assesses residents’ health from three dimensions—physiological, mental, and social health—the selection of indicators still has limitations, particularly for mental and social health. Due to measurement challenges, delayed monitoring timelines, and limited statistical data availability in China, it remains difficult to fully capture the specific conditions of residents’ mental and social health. Consequently, the range of available indicators is restricted. Relying solely on the emergency rate of psychiatric departments, the crude divorce rate, and the crime rate as proxies for mental and social health status may inevitably deviate from actual health conditions. The indicators selected in this study cannot comprehensively encompass the full scope of residents’ health, and the explanatory variables only partially reflect the impact of urban green space. Future research could adopt more diverse and micro-level indicators to more accurately evaluate both urban green space quality and residents’ health status.

## 5. Conclusions

Based on provincial panel data from 2007 to 2020 in China, we examined the relationship between urban green space and the comprehensive health status of residents using panel econometric models. It is generally agreed that urban green space promotes human health. Based on that statement, two main questions were answered in this study: (1) Does the influence of green space on residents’ health vary with differences in their health levels? and (2) Is the impact of green space on health related to the local climatic background? The results of this study have confirmed that the health-promoting effect of green spaces varies individually and regionally. If the inhabitants have a good state of health, green space has a significant health-promoting effect; if the inhabitants’ state of health is poor, their health level is not affected by green spaces. In addition, the response of green spaces to health varies between provinces with different climatic backgrounds. The health level and the per-capita park green area of the residents of mid-temperate and warm temperate regions are related, and increasing the park area in these regions can effectively improve the health status of the residents. The health level of residents in subtropical regions is not affected by the per-capita park green area. Therefore, it is necessary to develop reasonable urban green space planning strategies based on the health level and climatic background of residents in different provinces, especially in areas where residents have a better health status, and to further utilize the benefits of promoting health through urban green space.

## Figures and Tables

**Table 1:** Health-related indicators of residents.

Indicator	Variable	Definition
Life expectancy	*X*_1_	Mean length of life of a hypothetical cohort assumed to be exposed, from birth to death, to the mortality rates observed at a given year
Perinatal mortality	*X*_2_	Ratio of the number of stillbirths and deaths in the first week of life to the number of live births, where the perinatal period commences at 28 completed weeks of gestation and ends seven completed days after birth
Maternal mortality	*X*_3_	Ratio of the number of women who die each year from pregnancy or childbirth-related causes to the number of live births per 100,000 people
Incidence rate of Class A and B notifiable infectious diseases	*X*_4_	Incidence rate of Class A and B notifiable infectious diseases per 100,000 people
Morbidity rate of Class A and B notifiable infectious diseases	*X*_5_	Morbidity rate of Class A and B notifiable infectious diseases per 100,000 people
Number of medical outpatients	*X*_6_	Total number of outpatient visits, emergency visits, appointments, and other visits
Emergency rate of psychiatric departments	*X*_7_	Number of people with mental illness who seek treatment in psychiatric emergency departments of medical institutions per 100,000 people
Crude divorce rate	*X*_8_	Annual divorce count to total population ratio
Crime rate	*X*_9_	Proportion of the population that commits crimes per 100,000 people

**Table 2:** The factor analysis results of the KMO test and Bartlett’s test.

KMO Value		0.735
Bartlett’s Test of Sphericity	χ^2^	2573.487
	df	36
	Sig	<0.001

**Table 3:** Eigenvalues and cumulative variances for the components extracted by varimax rotated principal component analysis.

Component	Eigenvalues	% of Variance	Cumulative %
1	3.033	33.696	33.696
2	2.476	27.516	61.212
3	1.028	11.423	72.635

**Table 4:** Factor loadings of the variables in each component extracted by principal component analysis.

	Component 1	Component 2	Component 3
Life expectancy	−0.468	0.695	−0.102
Perinatal mortality	−0.149	0.755	−0.133
Maternal mortality	−0.380	0.672	−0.184
Incidence rate of Class A and B notifiable infectious diseases	−0.063	0.779	0.162
Morbidity rate of Class A and B notifiable infectious diseases	0.203	0.504	0.454
Number of medical outpatients	0.425	0.501	−0.009
Emergency rate of psychiatric departments	0.956	−0.227	0.041
Crude divorce rate	0.929	−0.070	−0.040
Crime rate	−0.085	−0.124	0.855

**Table 5:** Significance test of global spatial autocorrelation of CHI and PGA per capita.

Year	CHI			PGA per Capita		
	Moran’s I	Z-Score	*p* Value	Moran’s I	Z-Score	*p* Value
2007	0.052	1.310	0.190	0.256	3.728	0.000
2008	0.042	1.136	0.256	0.207	3.094	0.002
2009	0.079	1.574	0.116	0.189	2.884	0.003
2010	0.092	1.749	0.080	0.075	1.403	0.160
2011	0.125	2.087	0.037	0.121	2.043	0.041
2012	0.140	2.437	0.015	0.059	1.198	0.231
2013	0.157	2.595	0.009	0.119	2.139	0.032
2014	0.242	3.658	0.000	0.071	1.352	0.176
2015	0.222	3.385	0.000	0.122	2.057	0.039
2016	0.364	5.165	0.000	0.162	2.547	0.011
2017	0.397	5.610	0.000	0.128	2.110	0.035
2018	0.398	5.588	0.000	0.125	2.080	0.037
2019	0.430	5.980	0.000	0.052	1.120	0.263
2020	0.494	6.751	0.000	0.026	0.777	0.437

**Table 6:** Results of the FE, QRPD, and grouped regression model (dependent variable is CHI).

Variables	FE	QRPD Analysis					Grouped Regression		
		10%	30%	50%	70%	90%	Midtp	Warmtp	Subtropics
GPA per capita	0.113 ***	0.075	0.079 **	0.111 ***	0.141 **	0.147 ***	0.189 **	0.135 **	0.009
	(3.22)	(1.21)	(2.02)	(3.42)	(2.48)	(1.45)	(2.05)	(2.04)	(0.22)
GDP per capita	0.183 ***	0.188 *	0.051	−0.012	−0.035	0.057	−0.191	0.215 ***	0.427 ***
	(2.68)	(1.72)	(0.74)	(−0.21)	(−0.53)	(0.32)	(−0.89)	(2.51)	(5.28)
Medical	0.116 ***	0.146 **	0.077 *	0.133 ***	0.176 ***	0.289 **	0.383	0.158 ***	−0.059
	(2.75)	(2.06)	(1.71)	(3.58)	(2.72)	(2.49)	(1.28)	(3.38)	(−0.74)
Urbanization rate	0.169 ***	0.131 **	0.088 **	0.173 ***	0.183 ***	0.296 ***	−45.355 *	1.194 ***	0.489 **
	(5.31)	(2.01)	(2.12)	(5.06)	(3.07)	(2.76)	(−1.89)	(2.67)	(2.40)
Permanent population	−1.383 ***	−0.034	−0.214 ***	−0.284 ***	−0.331 ***	−0.428 ***	−0.970	−1.188 *	−0.181
	(−4.55)	(−0.39)	(−3.85)	(−6.18)	(−4.13)	(−2.97)	(−0.38)	(−1.78)	(−0.49)
Education	0.373 **	−0.248 **	0.006	0.021	0.055	0.152 **	0.231	0.024	0.091
	(5.83)	(−2.5)	(0.09)	(0.38)	(0.88)	(2.08)	(0.35)	(0.27)	(0.96)
R^2^	0.444						0.831	0.564	0.592

Notes: Z-scores are reported in parentheses, with * *p* < 0.1, ** *p* < 0.05, *** *p* < 0.01. GPA per capita stands for park green area per capita, Medical for number of medical and health institutions, Education for education expenditure per capita.

## Data Availability

The datasets used and analyzed during the current study are available from the corresponding author on reasonable request.

## Supplementary Materials

The following supporting information can be downloaded at https://www.mdpi.com/article/10.3390/healthcare13101109/s1, Supplementary Table S1. Factor score coefficient matrix. Supplementary Table S2. Results of multicollinearity diagnosis. Supplementary Table S3. Robustness checks using alternative variables.
