# Supplementary material for: A Longitudinal Empirical Study on the Association Between Urban Green Space Ratio and Population Health Indicators
Source: Healthcare (Basel). 2025 May 10;13(10):1109. doi: 10.3390/healthcare13101109 (PMC12111012; doi:10.3390/healthcare13101109)
Supplement: Supplementary file 1 [file healthcare-13-01109-s001.zip › healthcare-3601489-supplementary.pdf]

Supplementary Table S1. Factor score coefficient matrix.

	Component 1	Component 2	Component 3
Life expectancy	-0.060	0.253	-0.079
Perinatal mortality	-0.026	0.257	-0.163
Maternal mortality	0.077	0.336	-0.123
Incidence rate of Class A and B notifiable infectious diseases	0.102	0.363	0.162
Morbidity rate of Class A and B notifiable infectious diseases	0.147	0.277	0.436
Number of medical outpatients	0.070	0.343	-0.052
Emergency rate of psychiatric departments	0.315	0.047	0.000
Crude divorce rate	0.355	0.126	-0.080
Crime rate	-0.093	-0.074	0.840

Supplementary Table S2. Results of multicollinearity diagnosis.

**Variables**	**VIF**	**1/VIF**
GPApercapita	1.32	0.759
GDPpercapita	1.02	0.976
Medical	1.04	0.959
Urbanization rate	1.13	0.882
Permanent population	1.04	0.958
Education	1.36	0.733
**Mean VIF**	1.15	

Supplementary Table S3. Robustness checks using alternative variables.

Variables	FE	QRPD analysis					Grouped regression		
		10%	30%	50%	70%	90%	Midtp	Warmtp	Subtropics
GCBA	0.108***	0.072	0.082**	0.107***	0.145**	0.142***	0.195**	0.131**	0.011
	(3.18)	(1.19)	(2.05)	(3.38)	(2.51)	(1.42)	(2.08)	(2.01)	(0.24)
GDPpercapita	0.179***	0.182*	0.054	-0.014	-0.032	0.061	-0.187	0.208***	0.421***
	(2.65)	(1.70)	(0.76)	(-0.19)	(-0.50)	(0.34)	(-0.87)	(2.48)	(5.25)
Medical	0.119***	0.142**	0.079*	0.129***	0.172***	0.283**	0.375	0.162***	-0.062
	(2.78)	(2.03)	(1.73)	(3.55)	(2.70)	(2.47)	(1.26)	(3.35)	(-0.72)
Urbanization rate	0.165***	0.127**	0.091**	0.170***	0.179***	0.302***	-44.872*	1.176***	0.481**
	(5.28)	(2.03)	(2.15)	(5.03)	(3.05)	(2.78)	(-1.87)	(2.65)	(2.38)
Permanent population	-1.362***	-0.037	-0.208***	-0.291***	-0.324***	-0.415***	-0.956	-1.203*	-0.175
	(-4.52)	(-0.41)	(-3.82)	(-6.15)	(-4.10)	(-2.95)	(-0.37)	(-1.81)	(-0.47)
Education	0.368**	-0.241**	0.008	0.024	0.052	0.157**	0.225	0.028	0.095
	(5.80)	(-2.48)	(0.11)	(0.41)	(0.85)	(2.11)	(0.34)	(0.29)	(0.98)
R^2	0.439						0.827	0.558	0.587

Note: GCBA represents the green coverage rate of built-up areas.
